# Structural Dynamics of the Lipid Antigen-Binding Site of CD1d Protein

**DOI:** 10.3390/biom10040532

**Published:** 2020-04-01

**Authors:** Bruno Cuevas-Zuviría, Marina Mínguez-Toral, Araceli Díaz-Perales, María Garrido-Arandia, Luis F. Pacios

**Affiliations:** 1 Centro de Biotecnología y Genómica de Plantas (CBGP, UPM-INIA), Universidad Politécnica de Madrid (UPM)—Instituto Nacional de Investigación y Tecnología Agraria y Alimentaria (INIA), Campus de Montegancedo-UPM, 28223 Pozuelo de Alarcón (Madrid), Spain; bruno.czuviria@upm.es (B.C.-Z.); marina.mitoral@alumnos.upm.es (M.M.-T.); araceli.diaz@upm.es (A.D.-P.); maria.garrido@upm.es (M.G.-A.); 2 Departamento de Biotecnología-Biología Vegetal, Escuela Técnica Superior de Ingeniería Agraria Alimentaria y de Biosistemas (ETSIAAB), Universidad Politécnica de Madrid (UPM), 28040 Madrid, Spain

**Keywords:** CD1d, lipid antigens, CD1 molecules, molecular dynamics, electrostatic potentials

## Abstract

CD1 molecules present lipid antigens to T-cells in early stages of immune responses. Whereas CD1‒lipid‒T-cell receptors interactions are reasonably understood, molecular details on initial trafficking and loading of lipids onto CD1 proteins are less complete. We present a molecular dynamics (MD) study of human CD1d, the isotype that activates iNKT cells. MD simulations and calculations of properties and Poisson-Boltzmann electrostatic potentials were used to explore the dynamics of the antigen-binding domain of the apo-form, CD1d complexes with three lipid–antigens that activate iNKT cells and CD1d complex with GM2AP, a protein that assists lipid loading onto CD1 molecules in endosomes/lysosomes. The study was done at pH 7 and 4.5, values representative of strongly acidic environments in endosomal compartments. Our findings revealed dynamic features of the entrance to the hydrophobic channels of CD1d modulated by two α helices with sensitivity to the type of lipid. We also found lipid- and pH-dependent dynamic changes in three exposed tryptophans unique to CD1d among the five human CD1 isotypes. On the basis of modelled structures, our data also revealed external effects produced by the helper protein GM2AP only when it interacts in its open form, thus suggesting that the own assistant protein also adapts conformation to association with CD1d.

## 1. Introduction

The adaptive responses of human immune system against hazards associated to a large diversity of molecules crucially depends on their recognition to trigger signaling events. Antigen-presenting proteins and T-cell receptors (TCRs) are major players in this initial stage [1]. The recognition of peptide fragments originated at foreign or host proteins presented by major histocompatibility complex (MHC) class I and II molecules has long been known and is reasonably understood [1,2,3]. However, the recognition of lipid antigens is less well characterized. Lipid antigens are presented by cluster of differentiation 1 (CD1) molecules to TCRs for T-cell activation [4]. Although considerable advances have been made in recent years towards understanding the mechanisms of this activation mainly throughout the study of CD1-lipid-TCR interactions [5,6], significant gaps still remain in our knowledge about the initial loading of lipid antigens onto CD1 proteins.

On the basis of sequence homologies, the five existing isotypes of CD1 molecules labelled with a–e letters were organized into two groups. CD1 isotypes a–d are transmembrane proteins which make up group 1 and present lipids at cell surface to TCRs. The single member of group 2 is CD1e, an intracellular soluble protein [4,6] that could have a role as lipid transfer protein (LTP) [7]. Despite the similar structures, cellular pathways, and general modes of TCR-interactions, MHC, and CD1 molecules display differences because of the distinct physicochemical nature of peptides and lipids. These differences reflect in polymorphism (MHC proteins are polymorphic whereas CD1 proteins are non-polymorphic), distinct modes of gene regulation and population genetics (much simpler in CD1 proteins than in MHC proteins), and in the way in which MHC and CD1 molecules bind their targets. MHC and CD1 proteins share a similar architecture in which two large α-helices shape an antigen-binding groove with two channels. However, while in MHC proteins this groove is an open cleft at which peptides can remain largely exposed to solvent, in CD1 proteins it is similar to a large buried pocket with hydrophobic side chains lining the two channels (see below).

Differences in shape and size of the hydrophobic channels together with subtle differences in the portal entrance to the antigen-binding groove confer CD1 molecules the ability to discriminate lengths of hydrophobic chains as well as types of polar headgroups. These features allow CD1 proteins to present a great diversity of lipid antigens [4,6,8]. The crystal structures of a–d isotypes in complex with a variety of lipids (only one uncomplexed structure exists for CD1e [7]) reveal significant differences in the aperture of the portal entrance and in the volumes of the hydrophobic channels. While for a, b, and c isotypes there are few structures available in the Protein Data Bank (PDB: as of 21 March 2020, 9, 19, and 6, respectively), for CD1d there are 115 structures, 59 of them corresponding to complexes with TCRs. Two reasons explain the more abundant structural information on CD1d: the first one is that the TCRs of invariant natural killer T-(iNKT) cells are specific in recognizing foreign or self-lipids presented only by CD1d. These cells are key activators of immune responses to many important diseases such as cancer, autoimmune diseases, infection, allergy, etc. [9,10,11]. The second reason is that whereas humans express all isotypes, CD1d is the unique CD1 protein expressed also in mice (90 out of the 115 CD1d structures in the PDB are murine). Both reasons have motivated and helped to conduct much more research on CD1d-antigen-TCR interactions than on the remaining isotypes.

Since lipids are usually associated with membranes and CD1 proteins are unable to extract lipids from membranes, it is accepted that loading of lipid antigens onto CD1 molecules needs the assistance of helper LTPs. This loading occurs in three cellular locations: the endoplasmic reticulum (where CD1 molecules are assembled together with some lipids), the cell surface (where lipids loaded onto CD1 molecules can exchange to be presented to TCRs), and the compartments in the endosomal pathway [4,12]. Different types of lipids and LTPs are associated with these different locations but CD1 molecules load most of their lipid cargo for antigen presentation in endosomal compartments [4,12]. In this way, the relevant candidates to helper proteins reduce to the few LTPs known to localize to the endosomal pathway: saposins A–D, Niemann-Pick C2 protein, and ganglioside monosialic acid 2 activator protein (GM2AP), all of them proteins that show a pH-dependent activity [4,12]. The so far unique mechanistic proposal about lipid loading in endosomes was suggested in 2004 [13]. Dubbed “tug-of-war” model, it was based upon experimental results showing that saposins and GM2AP were able to unload lipid antigens already bound to CD1d. The model suggests that transient CD1d‒lipid‒LTP complexes should form and that CD1d and the LTP would then compete for the same lipid according to their respective affinities for it [13]. Although the “tug-of-war” model seems a sounding proposal, there is still no structural evidence supporting it. Given that lipid antigens are normally associated with membranes, and that their endosomal loading onto CD1 proteins occurs vesicles, the experimental study of the mechanistic details of this proposal is difficult. On the other hand, other LTPs such as fatty acid amide hydrolase (FAAH) are known to facilitate the presentation of some lipid antigens by CD1d [14]. Direct loading onto CD1d of α-GalCer (α-galactosylceramide) bound to FAAH with an activity equivalent to that of saposin B in lysosomes and extraction by FAAH of the bound glycolipid from CD1d have been demonstrated in vitro [14]. FAAH was also found to modulate the activity of α-GalCer in vivo through its serum transport [14]. Considering the different environmental conditions in endosomal compartments and in serum, the evidence gathered suggests that some intrinsic features of the CD1d molecule should be instrumental in lipid loading.

As mentioned above, crystal structures have provided essential information on interactions among CD1 isotypes, lipids and distinct TCR fragments. However, those structures represent a static picture of a molecular association and are subjected to crystal packing constraints that are not present in biological environments. Computational approaches appear thus as a valuable alternative to provide molecular details regarding the interactions among CD1 molecules, lipid antigens, and helper LTPs in subcellular compartments difficult to investigate with experimental biophysical/biochemical techniques. Molecular dynamics (MD) is a useful tool to study the dynamic evolution of molecular associations probing their changes with time and upon the influence of external factors. MD calculations were employed in 2013 to study the dynamics of lipid-free (apo-) and complexed (holo-) forms of human CD1 proteins [15]. This report presented MD results obtained with distinct force fields and programs for apo-forms of the five CD1 isotypes and single 100 ns simulations for one holo-form of each a-d isotype at protein–ligand geometries taken from their crystal structures [15]. MD simulations were also applied to explore states of CD1c in its apo-form and in two complexes with stearic acid and other small ligands [16] and to investigate how structural differences in mycolate lipids bound to CD1b impact the T-cell response in tuberculosis [17] However, the own process of lipid loading onto CD1 proteins is hard to address with all-atom MD calculations as it takes times well beyond the μs range [18]. Considering the large variety of the molecular systems involved in lipid loading, those times place this computational study in the current limits of MD simulations.

Aimed to elucidate molecular details that could shed light into intrinsic features of CD1 proteins relevant to lipid–antigen loading, we present an all-atom MD-based computational study at pH 7 (taken as reference) and at pH 4.5 (value representative of the strongly acidic media in late endosomes and lysosomes) of the following human CD1d systems. *(1)* Apo-form, *(2)* complexes with three lipid antigens known to be presented to TCRs of iNKT cells: α-GalCer, a potent inductor of type I iNKT-cell activation [4], LPC (lysophosphatidylcholine) presented by CD1d to a LPC-specific TCR of iNKT cells [19], and PHS (phytosphingosine) recognized by CD1d [20] and suggested to play a role as a moiety associated to iNKT activation in humans [21]. PHS is one of the two tails in α-GalCer and also one of the two molecular segments of the natural ligand of the major allergen from peach fruit Pru p 3 [22]. It has been demonstrated that PHS is presented by CD1d to iNKT cells playing an adjuvant role in promoting IgE sensitization to this allergen [23]. Additionally *(3)*, we also present results at both pH values for the complex of CD1d with the helper protein GM2AP in its open and closed forms bound to LPC and PC (phosphatidylcholine), respectively. Our study shows a pH-dependence of critical features of CD1d regarding the portal entrance and identifies key residues in the portal helices whose dynamic evolution depends on the loaded lipid and pH. Our data obtained with modelled structures of the CD1d-GM2AP complex also reveal the external effect produced by the helper protein GM2AP on those features only when it interacts with CD1d in its open form. This finding suggests that the own assistant protein should also adapt its conformation to the association with the CD1 molecule.

## 2. Materials and Methods

### 2.1. Initial Geometries of Lipid Antigens and Proteins

**Figure 1:**
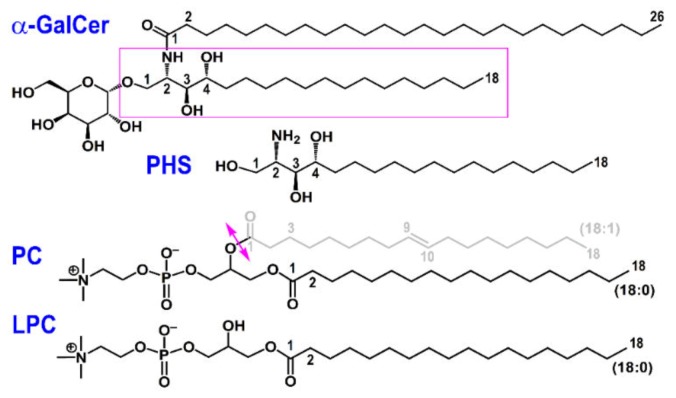
Lipid antigens considered in this study. The 18-C tail of α-GalCer (α-galactosylceramide) boxed in magenta is PHS (phytosphingosine). The hydrolysis of PC (phosphatidylcholine) at the bond indicated with the double magenta arrow yields LPC (lysophosphatidylcholine).

The lipids considered in this work are shown in Figure 1. Initial geometries of α-GalCer and LPC were taken from the crystal structures of human CD1d‒α-GalCer (PDB id 1ZT4 [24]) and CD1d‒LPC (PDB id 3U0P [19]) complexes. The initial geometry of PHS was taken from the final structure of our MD study of the peach allergen Pru p 3 in complex with its natural ligand and with PHS [23,25]. In the current work, LPC, which is the product of hydrolysis of PC as indicated in Figure 1, is also present as ligand in the open form of helper protein GM2AP (see below). In this case, the crystal structure of open GM2AP has a molecule of LPC and other of oleic acid (OLA) inside its hydrophobic cavity (PDB id 2AG4 [26]) whereas the crystal structure of closed GM2AP has a molecule of PC in the cavity (PDB id 2AG2 [26]). The crystal structures of these two complexes were used as initial geometries for our all-atom MD simulations of the CD1d-GM2AP systems presented below.

**Figure 2:**
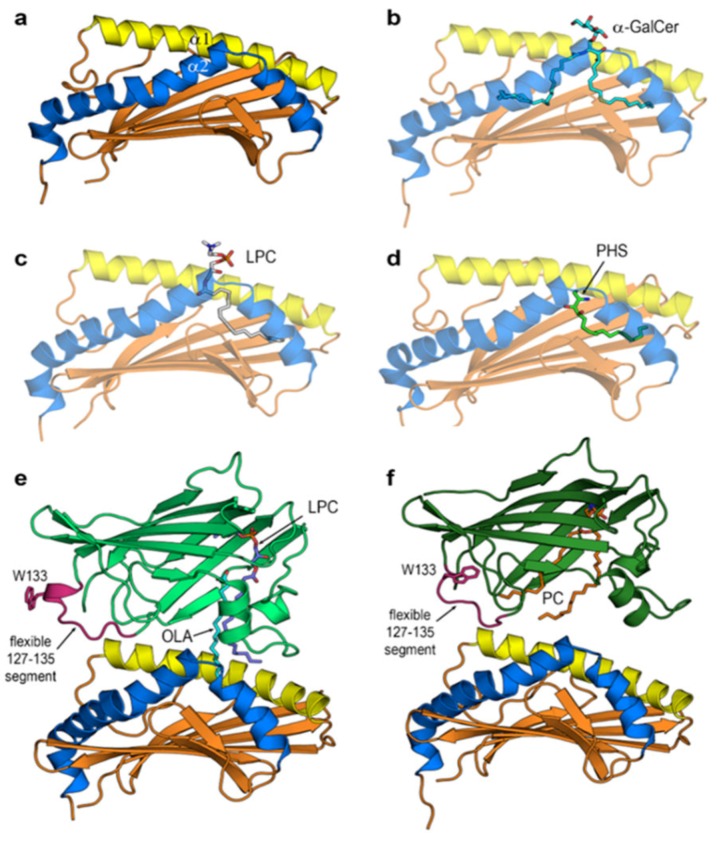
Initial geometries of CD1d systems studied. Only the antigen-binding α domain of CD1d shown was included in all-atom molecular dynamics (MD) calculations. α1 and α2 portal helices are colored yellow and blue, respectively. (**a**) Apo-CD1d. (**b**) CD1d‒α-GalCer complex. (**c**) CD1d‒LPC complex. (**d**) CD1d‒PHS complex. (**e**) Complex of CD1d and the open form of GM2AP (light green) with LPC and OLA. (**f**) Complex of CD1d and the closed form of GM2AP (deep green) with PC. These open and closed forms differ in the local structure of the flexible 127‒135 segment and in the conformation of W133.

**Figure 3:**
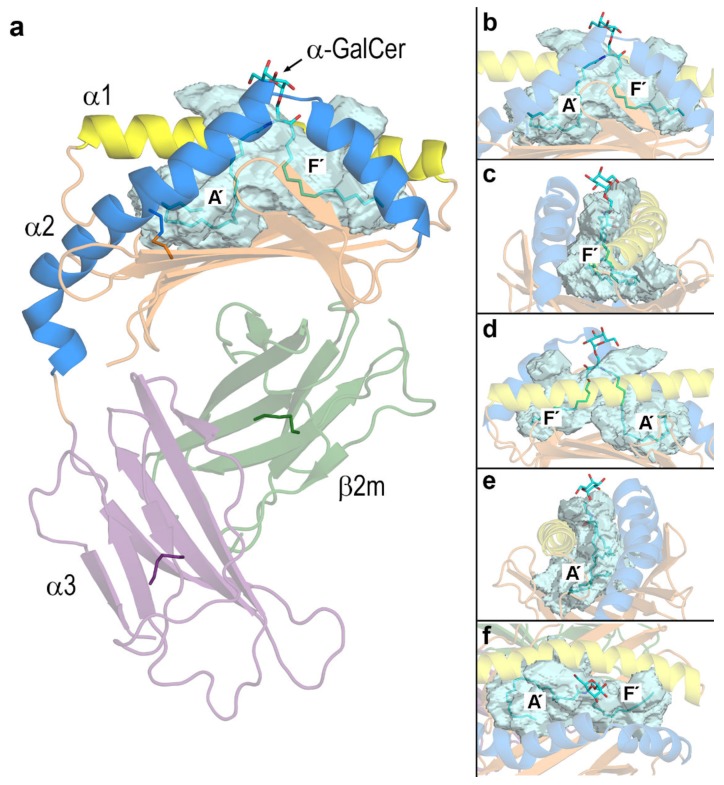
(**a**) Crystal structure of human CD1d‒α-GalCer complex (PDB id 1ZT4 [24]). The antigen-binding domain is composed of the α1 (yellow) and α2 (blue) helices that sit above a β sheet platform (orange). This α1+α2 segment is linked to the α3 domain (violet) that attaches CD1d to membranes through a transmembrane segment (not shown). The β2m domain (green) is linked non-covalently to the α domain. The pocket detected by DoGSite is shown as a transparent surface (cyan). A′ and F′ denote the two hydrophobic channels that are filled with the two tails of α-GalCer (sticks with carbons in cyan). (**b**)-(**e**) Zoomed views rotated 90° around a vertical axis and (**f**) top view of the portal.

For proteins, the initial geometries of CD1d were those of the two chains in the crystal structure of human CD1d‒α-GalCer complex (PDB id 1ZT4 [24]). The asymmetric unit of this structure has two molecules: one lipid-unbound and another lipid-bound. Whereas we used the former as initial setting for apo-CD1d, the latter was used as initial geometry for the remaining CD1d systems studied except for the CD1d-LPC complex whose initial setting was just that of its crystal structure (PDB id 3U0P [19]). The initial geometry of PHS in its complex with CD1d was obtained through protein–ligand docking (see Section 2.2). These settings gave the initial structures shown in Figure 2a–2d for apo- and holo-forms of human CD1d. The initial geometries of the open and closed forms of GM2AP were those of the above mentioned crystal structures 2AG4 and 2AG2 [26], respectively, while the initial geometries of their complexes with CD1d (Figure 2e,f) were obtained in protein–protein dockings as explained in Section 2.2. In all cases, only the antigen-binding α domain of CD1d (Figure 3) was included in the all-atom MD calculations.

### 2.2. Docking Calculations to Set Initial Geometries of Protein–Ligand and Protein–Protein Complexes

Protein–ligand docking calculations were performed with AudoDock Vina 1.1.2 [27] and the corresponding tool implemented in Chimera 1.13 [28] to obtain the initial geometry of PHS in the binding cavity of CD1d. The best pose (that having the lowest ΔG affinity energy) was selected as initial setting of the CD1d-PHS complex shown in Figure 2d.

The initial geometry of the CD1d‒GM2AP complex was obtained with protein–protein docking calculations in a three-step process: *(1)* Firstly, blind docking calculations were run with the following methods: ClusPro 2.0 [29], ZDOCK [30], and pyDock [31]. Among the 30 models settled by the 10 top solutions of every method, only two models showed an orientation of the binding sites of both proteins appropriate for possible lipid transfer. *(2)* These two models were then refined using them as input for (i) ZDOCK in user-defined binding segments mode [30], and (ii) HADDOCK 2.2 docking [32] which ask the user a list of interacting residues. *(3)* These refined solutions were then optimized with RosettaDock [33] and the highest-ranked solutions among the top 10 best-scored poses were finally selected as initial geometries shown in Figure 2e,f. Since all-atom MD calculations dynamically change the structures, docking calculations treating proteins as rigid bodies were used to set up the initial geometries in all cases.

### 2.3. Protonation States and Poisson-Boltzmann Electrostatic Potentials

Values of *pK_a_* for ionizable side chains were obtained separately for isolated CD1d and for CD1d in complex with GM2AP as well as for both the open and closed forms of GM2AP with the following methods: Propka 3.1 [34,35] H++ [36,37], and Rosetta-*pKa* [38]. *pK_a_* predictors apply empirical models to calculate ΔG for deprotonated and protonated states of Asp, Glu, His, Tyr, Lys, and Arg residues in their local environments in proteins, thus estimating deprotonation equilibrium constants. Since *pK_a_* values obtained in this way may involve considerable uncertainties, it seems advisable to employ more than one method and keep only similar results. We considered as similar those values which agree within ± 0.1 *pK_a_* units predicted by at least two out of the three methods. For the proteins and pH values dealt with here, this procedure allowed us to unambiguously assign *pK_a_* values to Asp and Glu residues. In the case of His, the three methods agreed in predicting very close values (differences smaller than 0.1 pK_a_ units). Acidic amino acids were then assumed to be protonated (0 charge) if their *pK_a_*’s are ≥ pH, and histidines were assumed to be protonated (+1 charge) if their *pK_a_*’s are ≤ pH. With protonation states thus assigned, input PQR files for computing Poisson-Boltzmann (PB) electrostatic potentials (EPs) were generated using Pdb2pqr [39,40]. Poisson-Boltzmann electrostatic potentials (PB-EPs) were obtained by solving numerically the nonlinear PB equation with the APBS 1.5 program [41] using the input interface implemented as a plug-in in PyMOL 2.3.2 [42]. Sequential-focusing multigrid APBS calculations were performed in 3D grids with 129^3^ = 2,146,689 points (step size ~0.5 Å) with dielectric constants of 4 for proteins and 78.54 for water at 0.150 M NaCl concentration. The numerical output of PB-EPs was saved in OpenDX scalar format for mapping onto molecular surfaces computed and rendered with PyMOL 2.3.2 [42].

### 2.4. Molecular Dynamics (MD) Calculations

All-atom 100 ns MD simulations were run for the six systems displayed in Figure 2 at both pH 7 and 4.5 using the July 2018 update of the CHARMM 3.6 force field [43] with c36m parameters for proteins [44]. These calculations were carried out with the high performance computing Power-MPI version of NAMD 1.12 [45] in the Magerit supercomputer of the Universidad Politécnica de Madrid. All systems were prepared with CHARMM-GUI [46] including ligand parametrization. Periodic solvation boxes with 15 Å spacing in all dimensions and TIP3P [47] water model were used and Na^+^ and Cl^−^ ions added to counter total charges and set 0.150 M salt concentration. The particle-mesh Ewald summation method was used for long-range electrostatic interactions and a 10 Å cutoff was set for non-bonded VdW interactions. Initial geometries shown in Figure 2 were minimized at 5000 conjugate-gradient optimization steps and water was then equilibrated at 298 K and 1 atm for 100 ps at 2 fs timesteps. Production runs were performed during 100 ns simulation time at 2 fs timesteps (50 million steps per simulation) in the NPT ensemble at 1 atm and 298 K with Langevin dynamics for T control and Nosé-Hoover Langevin piston method for P control. Output from NAMD was saved every 20,000 calculation steps thus rendering trajectories composed of 2500 frames that were processed and analyzed with Carma 1.7 [48] and VMD 1.9.3 [49].

### 2.5. Calculation of Molecular Properties

Pockets in the static structure of CD1d corresponding to the lipid-bound chain of the CD1d-α-GalCer complex (PDB id 1ZT4 [24]) were detected with DoGSite Scorer [50] whose output was processed with PyMOL 2.3.2 to render the images in Figure 3.

Changes along MD simulations of solvent-accessible surface area (SASAs), occupation of volumes defined by amino acids in the portal entrance, torsion angles of selected residues, and numbers of CD1d‒ligand hydrogen bonds were computed in the trajectories with Carma 1.7 [48] and VMD 1.9.3 [49] with the help of in-house Tcl/Tk scripts written to run in VMD. In some cases, plots of properties computed from 2500-frame trajectories were smoothed with Savitzky-Golay filtering using a 3^rd^-degree polynomial on windows composed of 51 frames.

Multiple structural alignments and calculation of scores measuring structural similarity were obtained with DALI [51,52]. For *N* structures being compared, DALI computes an *N*x*N* matrix of pairwise similarities and uses then heuristic techniques to compute optimized alignment scores.

All the molecular graphics were designed and rendered with PyMOL 2.3.2 [42].

## 3. Results

All the CD1d‒GM2AP data presented in this section are based on modelled initial geometries of the complex as no experimental structures exist for it.

### 3.1. Key Residues and pH-Dependent Electrostatics in the Portal Entrance to Lipid–Antigen Channels of CD1d

CD1 and MHC-I proteins are in charge of presenting lipid- and peptide-antigens, respectively, to specific TCRs to trigger immune responses. Both proteins share a similar structure composed of two non-covalently linked chains: the α chain which contains the antigen-binding domain and the β chain which consists of a β2-microglobulin (β2m) domain (Figure 3a). The α chain has in turn two well-separated domains: one defined by two large helices α1 and α2 that flank the antigen-binding cavity above a 6-stranded β sheet platform and other domain defined by an Ig-like β-sandwich domain (Figure 3a) which is however termed α3. This α3 domain attaches CD1 molecules to membranes by means of a transmembrane segment (not shown in Figure 3). The β2m domain is needed for the functional expression of CD1 proteins on the cell surface [4].

The lipid-antigen binding domain is thus defined by the α1 + α2 structural segment used in our study. MD simulations revealed that the α3 domain in the structurally similar MHC-I protein participates in peptide binding through an inter-domain twisting motion acting as an allosteric dynamic coupling [53]. Direct experimental evidence on this long-range dynamic communication has been found in some MHC-I allotypes [54]. However, this effect occurs upon interaction with the ER chaperone tapasin that influences MHC-I plasticity. Since our study is primarily aimed to address the features of CD1d relevant to lipid loading in endosomes and the number of CD1d systems here studied would render the MD calculations with the complete CD1d structure prohibitive, we used only the α1 + α2 structural segment in our MD study. This has been common practice in the preceding MD simulations on CD1 proteins [15,16,17] mentioned in the Introduction.

While α1 is a continuous helix with 8 turns (29 amino acids 60–88), α2 is composed of two helical segments connected by a small 2- or 3-residue hinge (this may change: see below) and spans about 44 amino acids (139–182). The portal entrance is defined mainly by amino acids 79 and 80 in the α1 helix and the hinge in the α2 helix. This structure shapes an antigen-binding cavity formed by the inner sides of the α1 and α2 helices and a floor settled by the β platform (Figure 3). This antigen-binding cavity features two large channels named as A′ and F′ that in the case of MHC-I proteins, remain exposed to the solvent while harboring the peptide antigen. In this case, the antigen-binding cavity should be more appropriately described as a cleft.

The lipidic nature of antigens presented by CD1 proteins makes this antigen-binding cavity to be largely buried. Similar to MHC-I molecules, the two channels in CD1 proteins (Figure 3) are also classified as A′ and F′ but their inner walls are lined with hydrophobic side chains. In the crystal structure of human CD1d‒α-GalCer complex (1ZT4 [24]), the ligand fits tightly in the cavity with the 26C-acyl chain (Figure 1) filling the A′ channel and the 18C-PHS tail filling the F′ channel (Figure 3).

The large pocket detected by DoGSite in CD1d in this complex covers also two small outer clefts to both sides of the portal entrance not occupied by the ligand. The galactose moiety of α-GalCer remains exposed at the portal entrance (Figure 3).

**Figure 4:**
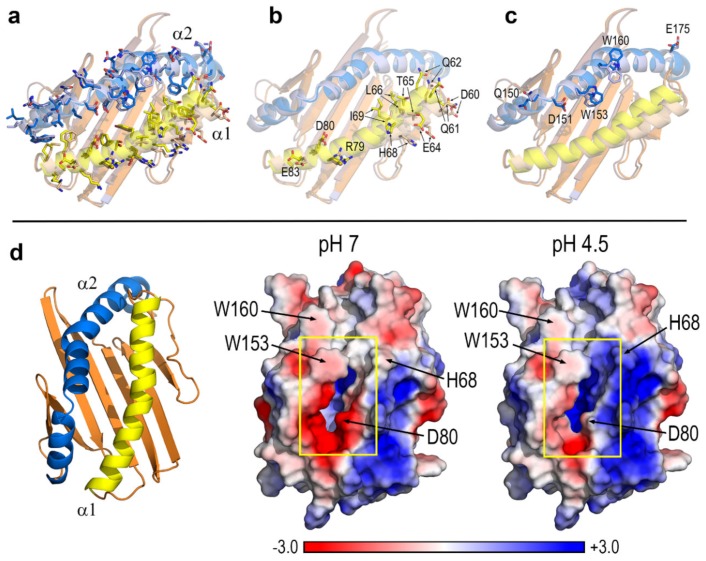
(**a**) Antigen-binding α domain of ligand-bound (yellow and blue) and ligand-unbound (light orange and light violet) chains in the crystal structure of human CD1d‒α-GalCer complex (1ZT4 [24]) showing all residues in the α1 and α2 helices. (**b**) Exposed residues either interacting with the lipid polar headgroup (79,80,83) or having significantly different side-chain conformation in the α1 helix. (**c**) Exposed residues either interacting with the lipid polar headgroup (151,153) or having significantly different side-chain conformation in the α2 helix. (**d**) PB-EP mapped onto the protein surface at pH 7 and 4.5 at the orientation shown on the left. Yellow boxes indicate the portal entrance to the cavity.

Although the authors of this crystal structure stated that human CD1d proteins do not refold in the absence of lipid ligands, they were able to obtain the geometry of a second ligand-unbound CD1d molecule [24]. During the preparation of crystals, the presence of more stable ligand-bound molecules apparently stabilized a population of non-ligand-bound molecules, and crystal packing requirements settled the position of both molecules in the crystallographic asymmetric unit [24]. The comparison of these two molecules allows to assess the effect of the ligand on the protein structure (Figure 4a–c). The superposition of both molecules from selection of the 29 amino acids in the α1 helix yields a RMSD for backbone atoms 0.82 Å whereas the superposition from selection of the 44 amino acids in the α2 helix (that shown in Figure 4a–c) yields an equivalent RMSD 0.64 Å. Hence, taking the α2 helix as reference, the superposition shows that the main difference regards the aperture of the groove caused by the more bent orientation of the α1 helix in the ligand-bound chain, a feature particularly noticeable in its first half (residues 60–70). In contrast, the α2 helix shows in the two molecules a more similar orientation concerning both the two segments and the hinge that characterize its geometry.

The superposition of the two molecules in the 1ZT4 structure shows two distinct features. On the one side, the bend of the α1 helix in the presence of ligand produces a shift in side-chains of residues 60–70. On the other side, the interaction with the exposed polar headgroup of α-GalCer provokes slight changes in the orientation of some residues at backbone segments that are nearly identical in the two molecules: Arg79 and Glu83 in the α1 helix, and Gln150, Trp153, and Trp160 in the α2 helix (Figure 4b,c). Of note, changes in side-chains of His68 and Trp160 are significantly greater than those observed in the remaining residues of the α1 and α2 helices. As discussed below, His68 is one of the residues that change their pKa at pH 4.5 and Trp160, in spite of being located far from the portal entrance, is one of the tryptophans found to behave as probes sensitive to external changes in the CD1d complexes studied (see Section 3.3).

At pH 7, the antigen-binding α1 + α2 domain has a net electric charge +1 arising from 17 acidic (7 Asp + 10 Glu) and 18 basic (9 Lys + 9 Arg) residues. At strongly acidic environments such as those existing in endosomes/lysosomes, some ionizable side chains change their protonation state producing a significant impact on the electrostatic features of this domain (Figure 4d). At pH 4.5, our estimates of pKa values predict protonation of Asp80, Glu132, Glu175, and the four histidines (sequence positions 38, 68, 105, and 115) yielding thus a net charge +8. However, the spatial distribution of the electrostatic changes occurring upon lowering the pH is the more interesting feature regarding possible effects on antigen-binding. Among the 7 residues protonated at pH 4.5, only 3 (His68, Asp80, and Glu175) are located in the portal helices and only His68 and Asp80 are close enough to the portal entrance so as to directly affect interactions with polar headgroups of lipid antigens. These two residues appear to be crucial in modifying the electrostatic nature of the surface in the close vicinity of the portal entrance from being clearly negative at pH 7 to markedly positive at pH 4.5 (Figure 4d). The α1 helix which is shown to bend in the ligand-bound molecule of 1ZT4 also participates, thus critically in modifying the electrostatic nature of the portal entrance at strongly acidic media.

### 3.2. Dynamic Changes in the Portal Entrance

**Figure 5:**
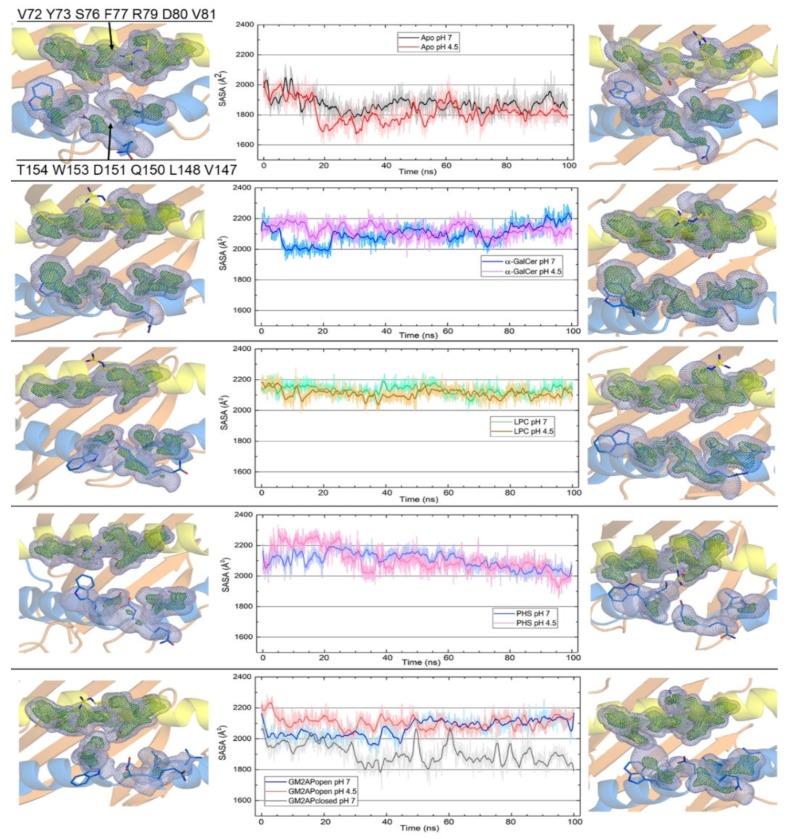
Occupancy volume grids (see the text) and solvent-accessible surface area (SASA) (plots) of residues in the α1 (yellow) and α2 (blue) helices flanking the portal entrance to the antigen-binding groove of CD1d computed over 100 ns all-atom MD simulations at pH 7 and 4.5 for the following systems (top to bottom): apo-form, α-GalCer complex, LPC complex, PHS complex, and complex with open and closed GM2AP. Inner grid (deep green) and outer grid (light blue) occupancy values are 0.9 and 0.5, respectively. These volumes are represented superimposed to final structures at pH 7 (left column) and 4.5 (right column). Residues defining the two flanking regions are indicated in the first inset. Ligands are omitted for clarity.

All-atom MD 100 ns simulations of apo- and holo-forms of human CD1d in the systems studied reveal dynamic changes in the portal entrance at both pH 7 and 4.5. Plots in Figure 5 show the variation along the simulation time of SASAs of residues flanking the portal entrance and grids display the occupation of space by those residues. This property is depicted as a grid volume defined in such a way that each grid point is set to 1 or 0 depending on whether it contains at least one atom or not. By treating atoms as spheres with VdW atomic radii, a grid point is considered to be occupied if it lies inside this sphere. When averaged over the frames of a trajectory, this gives the fractional occupancy of that grid point.

Occupancy values 0.5 (outer grids) and 0.9 (inner grids) shown in Figure 5 define space regions occupied 50% and 90% of simulation time by atoms of residues flanking the portal entrance, respectively. Lower values span broader volumes inside which most of the atoms in each residue are present most of the time. Therefore, only the more flexible segments of each side chain lie outside at times. Higher occupancy values span tighter volumes inside which only the more rigid segments (backbone and side-chain Cβ segments) are present most of the time. When represented superimposed to the final structures, those volumes leave the most mobile segments outside them. Residues such as Tyr73 and Arg79 in the α1 helix or Gln150, Asp151, and Trp153 in the α2 helix display the outer part of their side chains outside the 0.5 occupancy volumes thus revealing their higher mobility in most of the systems.

The information provided by the dynamic evolution of SASAs, and these occupancy volumes illustrate a key feature of this portal entrance: the extent of its aperture depends on (*i*) whether a ligand is harbored in the binding groove or not, (*ii*) the type of ligand, (*iii*) whether the helper protein in case of complex with CD1d has the proper conformation for interaction or not, and (*iv*) pH (Figure 5). The final structures of apo-CD1d show that occupancy volumes of the two flanking helical regions are close at pH 7 and even closer at pH 4.5, a result that indicates that the portal entrance remains nearly closed over the simulation. In addition, 0.5 and 0.9 volumes differ from each other in apo-CD1d. This result indicates that the outer segments of side chains (0.5 occupancy) move freely with respect to more rigid segments (0.9 occupancy) in the residues in the portal entrance, being this effect slightly more noticeably at pH 4.5 than at pH 7. The SASA of these two flanking helical regions decreases at the very beginning of the simulation and then remains at ~1900 Å^2^ although this decrease is overall smaller at pH 4.5. This result suggests that the portal entrance in CD1d closes immediately in the absence of ligand.

Occupancy volumes and SASAs for CD1d in the complexes with the three ligands suggest a dependence mainly on the type of polar headgroup (Figure 1). The final structures at both pH values of the complex with α-GalCer show two well separated occupancy volumes and a close resemblance between 0.5 and 0.9 occupancy volumes. These results indicate firstly that the portal entrance remains open along the simulation and, secondly, that outer side chain segments and inner segments of the residues in the two flanking regions move similarly during the simulation time. The SASA value of these regions remains near the initial value ~2200 Å^2^ along the simulation although it remains more steady at pH 4.5 than at pH 7. Similar results are found for the CD1d-LPC complex except that the evolution of SASA is now virtually identical at the two pH values and that the 0.5 and 0.9 occupancy volumes differ from each other in the α2 helix LPC and PHS differ from α-GalCer in that they have a single acyl chain whereas α-GalCer has two (Figure 1), but the three ligands are similar in that their polar headgroups remain largely exposed to solvent (Figure 2). In this way, the dynamic evolution of both the SASA values and occupancy volumes that directly probe the aperture of the portal entrance, suggests a sensitivity to the mere presence of a ligand segment (the polar headgroup) that remains exposed and not to the own nature of that segment. In fact, this polar segment is a voluminous sugar moiety in α-GalCer and an elongated chain with ammonium and phosphate groups in LPC or with amine and hydroxyl groups in PHS. In agreement with this, changes of occupancy volumes and SASA in the PHS complex show different features: close proximity between volumes and smaller areas, without significant differences between the two pH values. Of note, the space position of the side chain of Arg79 in the final structures of the PHS complex suggests that this residue may participate in ligand-binding. In contrast, the side chain of Arg79 orients far from the portal entrance in the complexes with α-GalCer and LPC.

As for SASA values and occupancy volumes of the portal entrance of CD1d in complex with GM2AP, results in Figure 5 suggest a noticeable dependence on structural features of the helper protein. The complex of CD1d with the closed form of GM2AP that bears a molecule of PC in its cavity (PDB id 2AG2 [26]: Figure 2) is unstable and dissociates at the beginning of the simulation (Figure S1). In sharp contrast, the complex of CD1d with the open form of GM2AP that bears one molecule of LPC and other molecule of OLA in its cavity (2AG4 [26]: Figure 2) is rather stable. GM2APopen shows in this complex a slightly greater mobility at pH 4.5 than at pH 7 whereas CD1d exhibits in all cases a low mobility, even in the dissociating complex with GM2APclosed (Figure S1). Interestingly, although CD1d has no ligand in its complex with GM2APopen, the presence of the GM2AP partner originates features of the portal entrance much more similar to any holo-form of CD1d than to its apo-form. In fact, the aperture of the portal entrance suggested by the separation between occupancy volumes as well as SASA values along simulations at both pH 7 and 4.5 resemble those of either CD1d‒α-GalCer or CD1d-LPC complexes and not those of apo-CD1d. Dissociation of the complex with GM2APclosed obviously leaves CD1d isolated and thus its SASA plot displays the same profile as that of apo-CD1d. However, the differences between the shapes of 0.5 and 0.9 occupancy volumes in the residues of the α2 helix suggest that the CD1d-GM2APopen interaction directly affects the α1 helix whose residues occupy similarly both volumes. In contrast, the outer side chain segment and the inner segments of the residues in the α2 helix move freely, suggesting they are not “constrained” by the interaction with GM2AP.

### 3.3. Dynamic Changes in Three Exposed Tryptophans of CD1d

The authors of the crystal structure of human CD1d‒α-GalCer complex noticed the different conformation of Trp40 and Trp63 inside the hydrophobic channels and the shift of exposed Trp140 and Trp160 when they compared the ligand-bound and ligand-unbound chains in that structure [24]. Before going into detail about dynamic changes of external tryptophans in the CD1d complexes here studied, it is illustrative to compare the five CD1 isotypes in search for differences in the contents of this amino acid. It must be considered that tryptophans are usually found in buried cavities frequently participating in π-staking interactions in hydrophobic core regions in proteins. If a tryptophan is exposed to solvent in a protein is also frequently found that it plays some role potentially relevant to the function of the protein [55,56,57,58,59]. The antigen-binding α1 + α2 domain of human CD1 isotypes have the following numbers of tryptophans: 7 in CD1a, 4 in CD1b, 5 in CD1c, 9 in CD1d, and 5 in CD1e. Trp residues at CD1d sequence positions 31, 40 and 53 (27, 36 and 49 in CD1e) are conserved in all isotypes, Trp23 (19 in CD1e) is present in all isotypes except CD1a, Trp131 (124 in CD1e) is present in all isotypes except CD1b, and Trp63 is only present in CD1a and CD1d. In addition, CD1a has also Trp14 and Trp51, and CD1d has also Trp140, Trp153, and Trp160, these three extra tryptophans having no equivalents in the remaining isotypes.

**Figure 6:**
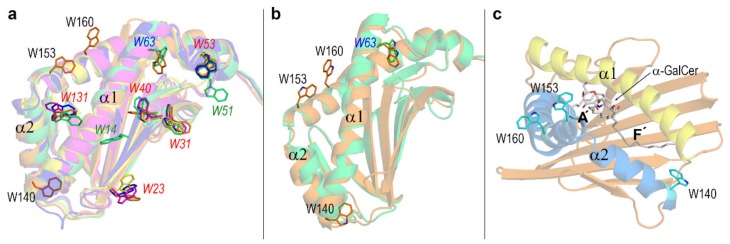
(**a**) Superposition of antigen-binding domains of the five human CD1 isotypes with the following crystal structures: CD1a PDB id 4X6E [60] (light green), CD1b PDB id 6D64 [61] (yellow), CD1c PDB id 6C15 [62] (blue), CD1d PDB id 1ZT4 [24] (orange), and CD1e PDB id 3S6C [7] (magenta). Side chains of all tryptophans in these domains are represented as sticks with the following labelling convention: red italics for Trp’s conserved in all CD1 isotypes, blue italics for Trp’s present only in CD1a and CD1d, green italics for Trp’s present only in CD1a, and black regular for Trp’s present only in CD1d. (**b**) Superposition of only CD1a and CD1d showing Trp’s located in the α helices. (**c**) Structure of CD1d‒α-GalCer complex (1ZT4) showing Trp’s located in the α2 helix and the ligand.

All the mentioned Trp residues are located in β strands except Trp63 (located in the α1 helix) in both CD1a and CD1d, and Trp140, Trp153, and Trp160 (located in the α2 helix) only in CD1d (Figure 6a: the structures deposited most recently in the PDB with the references indicated in the caption to Figure 6 are used for human CD1a, CD1b, and CD1c). Among the three tryptophans strictly conserved in all isotypes (31, 40, and 53 in CD1d), only one (Trp40) is involved in lipid-binding. This residue acts as a lid restricting the space available to acyl chains of lipid antigen in the cavity [24]. As for tryptophans present in all except one isotype (23 and 131 in CD1d), Trp23 is oriented towards the β2m domain whereas Trp131 is involved in lipid binding forming part of the inner wall inside the cavity. Trp14 and Trp51, only present in CD1a, have different structural roles as the former has its side chain interacting with the lipid in the hydrophobic groove while the latter is oriented towards the α3 domain. The conclusion of this comparative analysis is that among the five CD1 isotypes, only CD1d has tryptophans in the portal helices (Figure 6a) and besides, three of them (140, 153, and 160) are exposed to solvent Although Trp63 (also present in CD1a) and Trp140 are too far from the portal entrance to expect them to directly participate in lipid-binding, Trp153 and Trp160 are in the close neighborhood of that entrance (Figure 6b,c).

**Figure 7:**
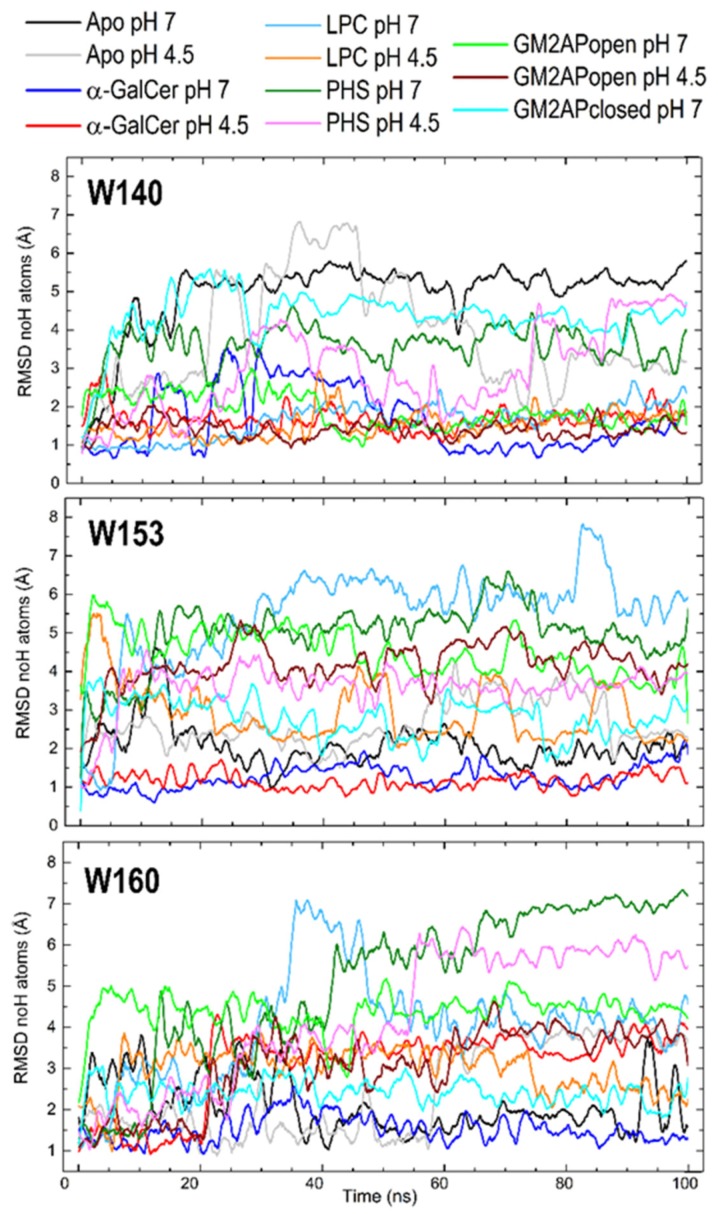
RMSD computed for non-hydrogen atoms of Trp140, Trp153, and Trp160 in all-atom MD 100 ns trajectories for the apo form and all the complexes of CD1d studied.

Trp140 is located too far from the portal entrance for being able to directly interact with ligands although it is located at the surface of the end of the F′ channel (Figure 6c). However, our MD simulations show that Trp140 is sensitive to the presence of a harbored ligand (Figure 7), probably due to the external surface changes associated with internal adjustment of the F′ cavity upon antigen binding. The side chain of Trp140 exhibits a considerable mobility in the apo form at both pH values and in isolated CD1d left behind after dissociation of the CD1d-GM2APclosed complex, but also in the CD1d‒PHS complex. In all these cases, the F′channel is either empty or occupied by a short segment of PHS. In sharp contrast, Trp140 in the CD1d complexes with α-GalCer and LPC and, which is more remarkable, in the complex with GM2APopen shows very low RMSD < 2 Å, particularly after ~50 ns (Figure 7) at the two pH values. This result suggests that the presence of a lipid chain filling the F′ channel induces the abovementioned external surface changes at the Trp140 region.

Trp153 is located just at the portal entrance (Figure 6) and the RMSD values of its side chain display a far greater dispersion than those of Trp140 or Trp160. Large values > 5 Å are found in the LPC and PHS complexes at pH 7. Both lipid antigens are characterized by a single acyl chain and exposed polar headgroups that move considerably along the simulation. On the contrary, small RMSD values < 2 Å are shown by the apo form and still smaller RMSD values ~ 1 Å are shown by the α-GalCer complex, irrespective of pH in both cases. The particularly low mobility of Trp153 in these two cases might be due to the close proximity of groups that restrict movement of its side chain. In apo-CD1d, these groups are the side chains of the α1 helix (see Figure 5) and in the α-GalCer complex it is the presence of the voluminous galactose moiety that remains at a fixed position in space because of the geometry imposed by the two long acyl chains that nearly completely fill the two cavity channels.

Trp160 shows RMSD curves that reach an equilibrium after ~40–50 ns separating into three groups: greater values > 5 Å for the CD1d‒PHS complex, smaller values < 2 Å for apo-CD1d, the isolated state after dissociation of complex with GM2APclosed and CD1d-α-GalCer complex (the three at pH 7), and intermediate values between ~2.5 and 4.5 Å for the remaining systems (Figure 7). Given that Trp160 is located in the middle of one of the two segments that make the α2 helix (Figure 6) far from the portal entrance where the headgroups of ligands are exposed, the mobility of its side chain must in turn be determined by changes in the helix α2 upon complexation (see Section 3.4).

**Figure 8:**
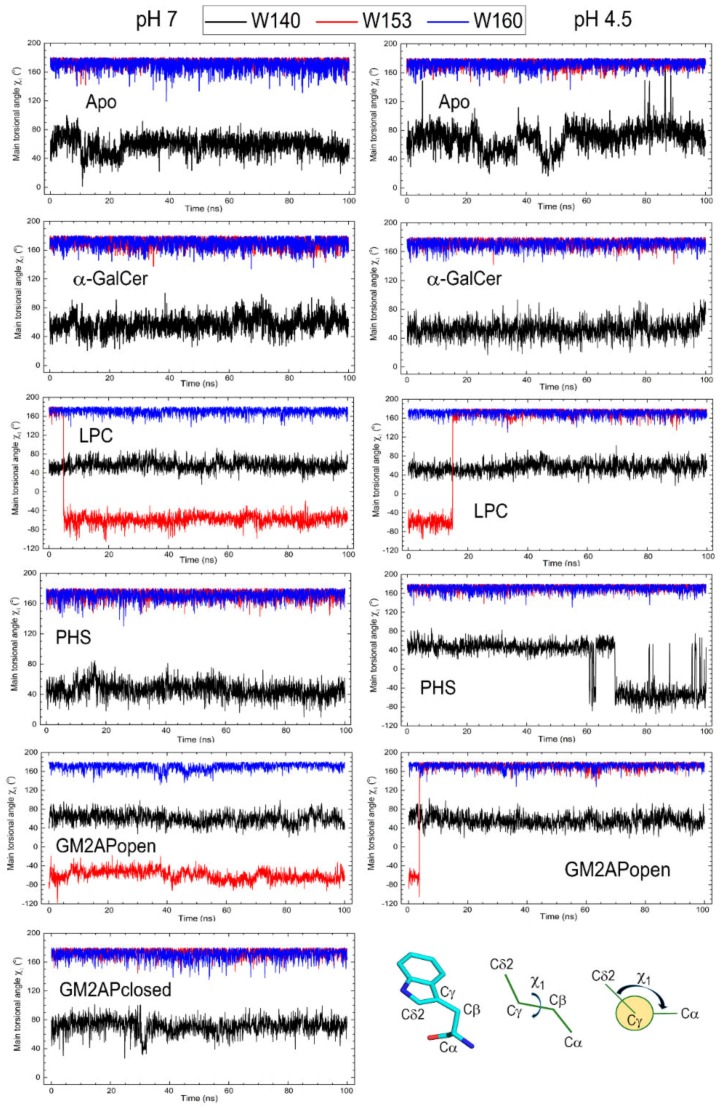
Changes of the main torsional angle χ_1_ defined at bottom right for tryptophans 140, 153, and 160 in CD1d computed along all-atom MD 100 ns trajectories for all the CD1d systems studied in this work. Plots in the left column correspond to pH 7 and plots in the right column to pH 4.5.

We studied the side chain torsions of Trp140, Trp153, and Trp160 by computing the changes of the rotamer-defining main torsional angle χ_1_ along all-atom MD 100 ns simulations. Setting of χ_1_ angle and plots of these changes are displayed in Figure 8. Mean values of this angle together with their standard deviations computed along those trajectories are shown in Table 1. Except where noted below, Trp140 has a dominant *g+* rotamer (χ_1_ = 60°) whereas both Trp153 and Trp160 have *trans* rotamers (χ_1_ = 180°).

Trp140 is the tryptophan showing the by far larger torsional fluctuations, particularly in apo-CD1d at pH 4.5 and in the CD1d-PHS complex also at pH 4.5 in which the rotamer *g+* changes to *g-* (χ_1_ = −60°) at about 60 ns (Figure 8). Trp153 side chain conformation is near the trans rotamer with little fluctuations (standard deviations around 6°) except in CD1d‒LPC and CD1d‒GM2APopen complexes in which changes between *trans* and *g-* rotamers are found. However, after these changes the side chain of Trp153 exhibits a steady small fluctuation similarly to that observed in systems in which no rotamer change is observed (Table 1 and Figure 8). Trp160 side chain conformation is rather similar in all systems with a *trans* rotamer kept along the simulation and little deviations about 7° except in the apo form at pH 7, in which a significantly larger fluctuation amounting to a standard deviation of nearly 18° is found (Table 1 and Figure 8).

To summarize this subsection, CD1d is the only CD1 isotype that has three tryptophan residues located in the α2 helix completely exposed to solvent. Although they do not participate directly in interactions with the lipid antigen, these three tryptophans display a dynamic behavior sensitive to the type of ligand harbored in the hydrophobic channels and to the type of polar headgroup exposed at the portal entrance. Even though no ligand is transferred from the helper protein to CD1d in our study, those three tryptophans show dynamic features in the CD1d‒GM2APopen complex similar to those of the CD1d‒lipid antigen complexes. Of note, pH seems to impact especially on Trp140 and Trp153 (Table 1 and Figure 8).

### 3.4. Final Structures of CD1d after MD Simulations

**Figure 9:**
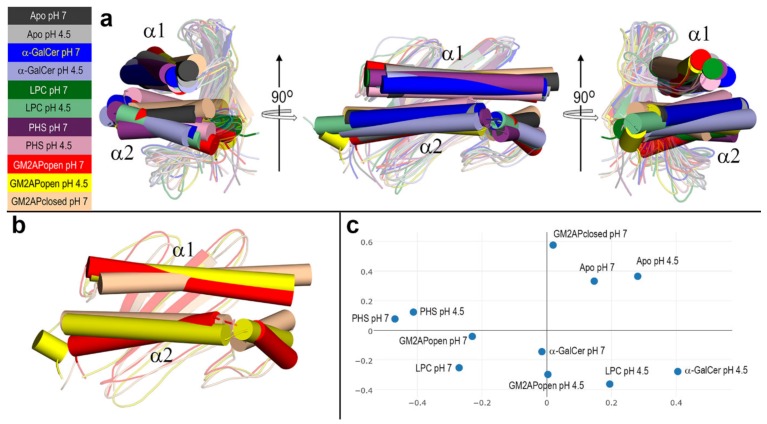
(**a**) Multiple structural alignment obtained with DALI of the final structures of CD1d after all-atom MD 100 ns simulations. Views are obtained by a counterclockwise 90° rotation around a vertical axis and the portal helices α1 and α2 are depicted as cylinders. (**b**) Structural alignment of the final structures of CD1d in the three complexes with GM2AP. Same color code as that used in (**a**). (**c**) DALI correspondence analysis of the multiple structural alignment.

Final structures of CD1d obtained after all-atom MD 100 ns simulations were compared by performing a multiple structural alignment with DALI [51,52]. For *N* structures being compared, DALI computes an *N*x*N* matrix of pairwise similarities using some heuristics to compute optimized alignment scores. DALI then applies a multidimensional scaling method to obtain a correspondence analysis, thus providing a representation in the form of a 2D plot in which data points of more similar structures are positioned near each other. Results of the multiple alignment of the final structures of CD1d after all-atom MD 100 ns simulations are shown in Figure 9. Ends of the portal helices determined with DSSP [63] in these final structures are given in Table 2.

The superposition of the final structures reveals considerable differences in the aperture of the two portal helices produced by different orientation of the α1 helix and different bending and orientation of the two (or three in CD1d‒GM2APopen complex at pH 4.5) segments that make α2 helix (Figure 9a). This high plasticity of the helices that form the antigen-binding domain of CD1d suits not only the type of ligand harbored in the hydrophobic channels but is also a pH effect. This is clearly noticed in the distinct geometry adopted by both helices in apo-CD1d at pH 7 and 4.5 (Figure S2). While the α1 helix is invariably defined by residues 60–88, the two segments that form the α2 helix vary their limits, much more noticeably in the second segment (Table 2). This implies that the hinge between segments in the α2 helix also changes from being composed of just one single amino acid (151) to a maximum of three (150–152). In the CD1d‒GM2APopen complex at pH 4.5, DSSP identifies an additional break in the α2 helix that is thus defined by three segments (Table 2 and Figure 9b). These results indicate that not only the type of lipid antigen but also the presence of the GM2AP helper protein (even in the absence of ligand inside CD1d), affect the spatial geometry of the portal helices (Figure 9b).

The correspondence analysis reveals interesting relationships (Figure 9c). For example, the final structures of the apo forms at both pH values as well as that of the isolated CD1d left after breaking of its complex with GM2APclosed, are positioned together. Except CD1d in the PHS complex at pH 4.5, the final structures in all the complexes at pH 7 are grouped together. Additionally, a similar result is found for CD1d in all the complexes at pH 4.5, except now the α-GalCer at pH 7. Note also that the three final structures of CD1d corresponding to the complexes with GM2AP are clearly separated (Figure 9c). These results suggests a more significant structural change occurring in a complex of CD1d with other protein than that occurring in the complexes with ligands as different as those investigated here.

## 4. Discussion

MD is a well-established approach to study the time evolution of biomolecular structures and interactions thus providing mechanistic details on a variety of processes. MD seems particularly useful to address systems involving lipidic molecules in cellular compartments such as endosomal vesicles that are hard to study experimentally. However, the time scale of lipid loading onto proteins is estimated to be at the µs range [18]. Considering the diversity and size of the molecular systems involved, in practice it is also difficult to address computationally in full this process. Notwithstanding, MD can be used to obtain information on CD1 proteins in charge of presenting lipid–antigens to TCRs that could be of relevance to understand lipid-loading from a dynamic point of view.

Along this line, we have studied by means of all-atom MD 100 ns simulations the apo-form of CD1d and its complexes with three lipid antigens (α-GalCer, LPC, and PHS) and with GM2AP, a protein known to assist lipid-loading onto CD1d in endosomes [4,12]. GM2AP has a large hydrophobic cavity and a high degree of dynamics that is reflected in an open and a closed form produced by lateral movements of a flexible segment (Figure 2e,f). In this way, GM2AP controls the size of the pocket opening and the width of the hydrophobic cleft depending on the lipid to be bound [26]. After exploratory protein–protein dockings, selecting the structures that predicted a proper orientation of the pocket in GM2AP with the portal entrance in CD1d, and refining these latter structures, we obtained an initial geometry of this complex suited to possible lipid transfer (Figure 2). MD simulations on all these CD1d systems were performed at pH 7 as reference value and at pH 4.5 as a representative value of the strongly acidic media in endosomal and lysosomal compartments.

Our results reveal that the protonation of seven amino acids in the antigen-binding domain of CD1d produces a significant variation of the electrostatic nature of protein surface. This variation affects particularly to the portal entrance to the hydrophobic channels of CD1d that changes from strongly negative at pH 7 to strongly positive at pH 4.5. This change is all the more remarkable since only two (His68 and Asp80 located in the α1 helix) out of those seven residues protonated at pH 4.5 are close enough to the entrance. The effect of acidic pH seems crucial to electrostatically select the nature of molecular partners of CD1d in endosomal compartments. Thus, it seems possible to modify the interactions with lipid antigens and with other proteins by just protonating a few specific residues. However, lipid exchange and antigen presentation to TCRs of iNKT cells occur in cell surface [4,12], which indicates that CD1d is also able to load lipids at physiological pH. The efficacy of this loading for interactions with iNKT cells has been demonstrated in vitro with the help of different surfactants using CD1d molecules from different species [64]. The available evidence on lipid-loading in the endosomal pathway and on lipid exchange in cell surface indicate that CD1d is able to modulate its properties to suit lipid trafficking requirements depending on the cellular localization.

Dynamic changes along MD simulations of occupied volumes and SASAs of residues in the close neighborhood of the portal entrance show that the aperture of this site mainly depends on whether a ligand is present in the cavity or not and on the type of ligand. Both geometrical descriptors indicate that CD1d closes its cavity at the immediate beginning of simulation in the absence of ligand. However, despite the frequent claims in the literature that empty CD1 proteins “collapse”, our MD results indicate that this closure is not accompanied by dynamic destabilization of apo-form. In holo forms, these dynamic changes depend essentially on the type of polar headgroup of the ligand exposed to solvent. In the case of GM2AP complexes, our MD results reveal that the complex with the closed form of GM2AP dissociates almost immediately in the simulation while the open form of GM2AP forms stable complexes. Interestingly, even though CD1d has no ligand in the complex with GM2APopen, the presence of the GM2AP partner originates features of the portal entrance much more similar to any holo-form of CD1d than to its apo-form. Structurally, all these changes are ultimately produced by slight bending and displacement of the portal helices.

The comparison of the five human CD1 isotypes shows that their antigen-binding domain has five tryptophans conserved in all or all except one isotypes. These tryptophans are located in β-strands and some of them participate in ligand-binding interacting with acyl chains in the hydrophobic channels of the CD1 groove. However, CD1d is unique in having three additional tryptophans at sequence positions 140, 153, and 160 located in the α2 helix, without equivalent in any other CD1 isotype. Furthermore, these three tryptophans have their side chain completely exposed to solvent. Tryptophan is an aromatic amino acid expected to appear in buried regions in proteins. When exposed to solvent, it usually plays an important role in structural stability or in interactions relevant to the function of the protein. The finding that CD1d is the only CD1 isotype with three exposed tryptophans in the α2 helix could point to a putative role either in lipid loading or in antigen-presenting processes. Our dynamic analysis showed that although Trp140, Trp153, and Trp160 do not directly interact with lipid antigen, they are sensitive to *(a)* the type of ligand in the hydrophobic cavity, *(b)* the type of polar headgroup exposed to solvent at the portal entrance, *(c)* the presence of helper GM2AP even though no ligand is transferred from GM2AP to CD1d in the complexes studied, and *(d)* pH, especially in Trp140 and Trp153.

The MD study presented in this work indicates that the main effect of the dynamic evolution of CD1d structure is the distinct inner space left between the two portal helices depending on the type of lipid antigen and pH (Figure 9). This space is controlled by the orientation of the α1 helix as well as by the bending and orientation of the segments that make the α2 helix. Our results showed that while the α1 helix is invariably composed of residues 60–88, the segments that define the α2 helix and the hinge between them change depending on the ligand and pH. Furthermore, the presence of a partner protein such as GM2AP investigated here provokes the re-arrangement of the portal helices so that CD1d in complex with this helper protein shows features much more similar to those of any holo-form than to those of the apo-form, even in the absence of lipid antigen harbored in the cavity of CD1d.

Despite these ligand- and pH-dependent dynamic changes, the multiple structural alignment of the final structures of CD1d obtained after 100 ns MD simulations shows similarities between the apo-forms at the two pH values considered, among the complexes at pH 7, and among the complexes at pH 4.5. This comparison analysis also reveals that the final CD1d structures resulting from the different complexes with the helper GM2AP protein show less similarities.

Summarizing, our computational study of CD1d, a protein in charge of presenting lipid antigens to T-cell receptors of iNKT cells with a paramount importance in the immune responses to major diseases, provides structural details of interest to understand binding mechanisms of CD1 molecules.

## 5. Conclusion

The molecular dynamics study of the lipid-presenting CD1d molecule in its apo-form and in complexes with three lipid–antigens and with GM2AP, a protein known to assist lipid-loading in endosomal compartments, revealed essential features of the antigen-binding domain. The high degree of plasticity of two α helices in this domain regulates the aperture of the portal entrance to hydrophobic channels that harbor lipidic acyl chains. Our results show a dynamic sensitivity of these helices to the type of lipid antigen, to the presence of a partner protein and to pH effects. This sensitivity is also shown by three exposed tryptophans in one of those two helices unique to CD1d among human CD1 isotypes. All these dynamic features might play a key role in lipid trafficking processes, in which CD1 molecules participate in earlier stages of triggering immune responses.

## Figures and Tables

**Table 1:** Mean values of the main torsional angle χ_1_ and standard deviations (in parentheses) of Trp140, Trp153, and Trp160 computed along all-atom MD 100 ns simulations for the CD1d systems studied. Values in italics correspond refer to trajectories in which a change of rotamer occurs ^1^.

CD1d System	Trp140	Trp153	Trp160
Apo form pH 7	58.4 (12.1)	173.8 (4.8)	167.4 (17.7)
Apo for pH 4.5	69.0 (16.6)	173.0 (5.3)	172.2 (6.1)
α-GalCer pH 7	57.3 (11.6)	170.9 (6.5)	169.9 (7.4)
α-GalCer pH 4.5	52.7 (10.9)	172.7 (5.4)	170.7 (6.4)
LPC pH 7	56.4 (11.0)	*−47.1 (50.8)*	170.5 (7.0)
LPC pH 4.5	55.8 (11.4)	*133.3 (87.0)*	171.2 (6.8)
PHS pH 7	46.1 (10.1)	171.8 (6.2)	170.6 (6.6)
PHS pH 4.5	*16.0 (49.4)*	172.6 (5.8)	170.5 (7.3)
GM2APopen pH 7	60.4 (12.5)	−59.5 (11.7)	169.7 (7.8)
GM2APopen pH 4.5	56.2 (12.8)	*163.4 (44.9)*	171.4 (6.9)
GM2APclosed pH 7	70.9 (11.2)	173.3 (5.3)	170.6 (7.1)

^1^ Plots for all the trajectories used to compute values in this table are shown in Figure 8.

**Table 2:** Identification determined with DSSP of sequence positions that define α1 and α2 helices in final structures of CD1d after all-atom MD 100-ns simulations in the systems studied.

CD1d System	α1	α2
Apo form pH 7	60‒88	139‒150 + 153‒177
Apo for pH 4.5	60‒88	139‒150 + 152‒181
α-GalCer pH 7	60‒88	139‒150 + 152‒176
α-GalCer pH 4.5	60‒88	139‒149 + 152‒181
LPC pH 7	60‒88	139‒150 + 153‒181
LPC pH 4.5	60‒88	139‒150 + 153‒182
PHS pH 7	60‒88	139‒150 + 152‒177
PHS pH 4.5	60‒88	139‒149 + 153‒176
GM2APopen pH 7	60‒88	139‒149 + 153‒182
GM2APopen pH 4.5	60‒88	139‒150 + 152‒176 + 179‒181
GM2APclosed pH 7	60‒88	139‒150 + 152‒178

## Supplementary Materials

The following are available online at https://www.mdpi.com/2218-273X/10/4/532/s1, Figure S1: RMSD of CD1d‒GM2AP complexes. Figure S2: Structural alignment of final structures of CD1d‒lipid complexes.
