# Supplementary material for: Structural Dynamics of the Lipid Antigen-Binding Site of CD1d Protein
Source: Biomolecules. 2020 Apr 1;10(4):532. doi: 10.3390/biom10040532 (PMC7226365; doi:10.3390/biom10040532)
Supplement: Supplementary file 1 [file biomolecules-10-00532-s001.pdf]

Supplementary Materials

Structural Dynamics of the Lipid Antigen-Binding Site of CD1d Protein

Bruno Cuevas-Zuviría [1], Marina Mínguez-Toral [1], Araceli Díaz-Perales [1,2], María Garrido-Arandia [1], and Luis F. Pacios [1,2,*]

[1] Centro de Biotecnología y Genómica de Plantas (CBGP, UPM-INIA), Universidad Politécnica de Madrid (UPM) – Instituto Nacional de Investigación y Tecnología Agraria y Alimentaria (INIA), Campus de Montegancedo-UPM, 28223 Pozuelo de Alarcón (Madrid), Spain ; bruno.czuviria@upm.es; marina.mitoral@alumnos.upm.es; araceli.diaz@upm.es; maria.garrido@upm.es

[2] Departamento de Biotecnología-Biología Vegetal, Escuela Técnica Superior de Ingeniería Agraria Alimentaria y de Biosistemas (ETSIAAB), Universidad Politécnica de Madrid (UPM), 28040 Madrid, Spain;

* Correspondence: luis.fpacios@upm.es

	Page
Figure S1	S2
Figure S2	S3

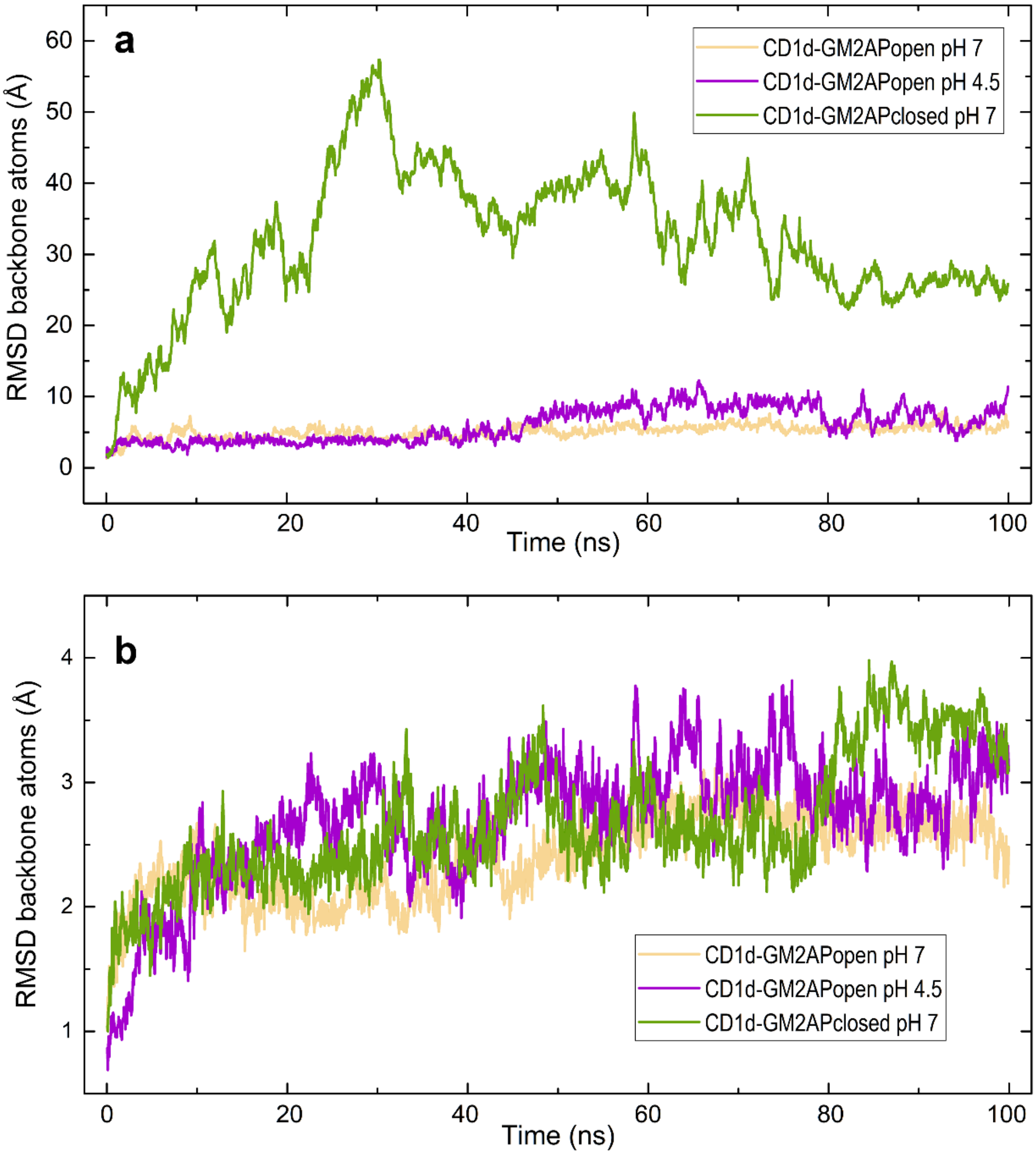

Figure S1. RMSD computed with backbone atoms along 100 ns all-atom MD simulations of CD1d-GM2AP (open form)–LPC & OLA complex at pH 7 and 4.5 and CD1d-GM2AP (closed form)–PC complex at pH 7. (**a**) RMSD of the complex with both proteins included in the calculation. (**b**) RMSD of only CD1d protein in the complex.

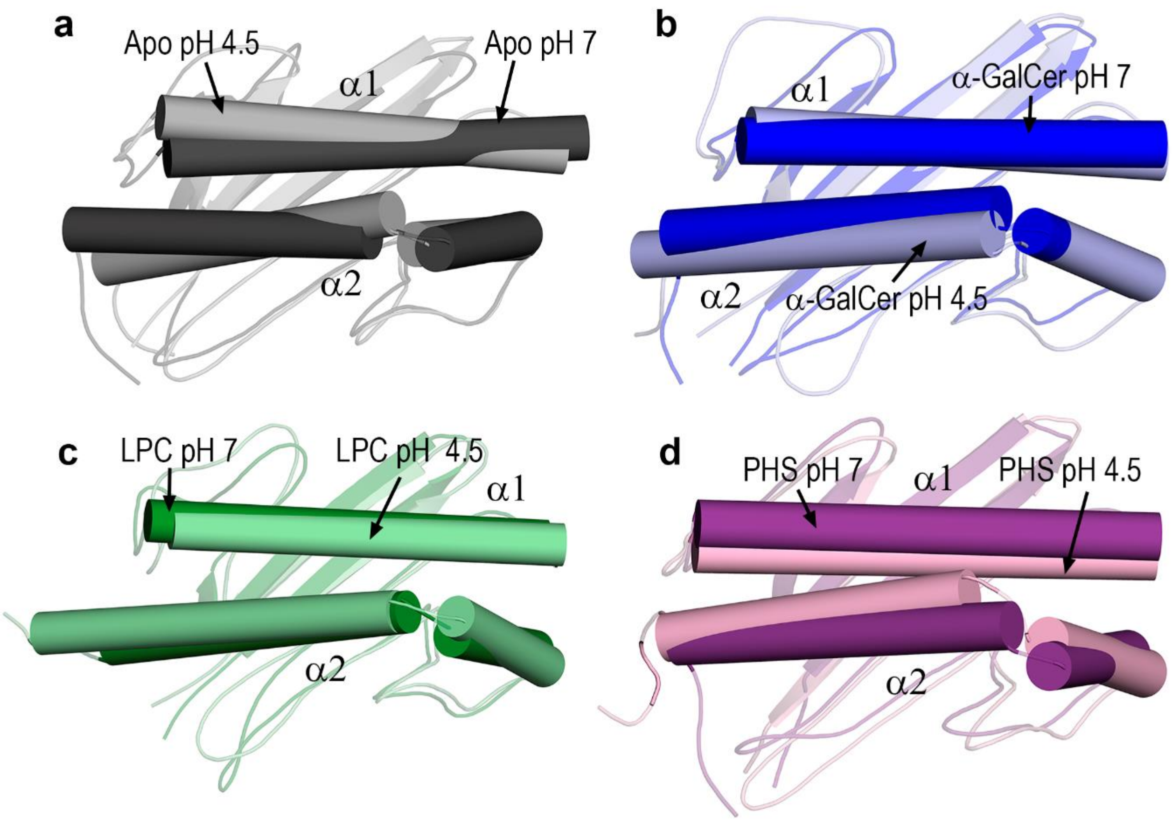

Figure S2. Structural alignment obtained with DALI of the final structures of CD1d after all-atom MD 100 ns simulations at pH 7 and pH 4.5 for (**a**) apo-form, (**b**) complex with α-GalCer, (**c**) complex with LPC, and (**d**) complex with PHS.
